# Impact of Human Immunodeficiency Virus Type-1 Sequence Diversity on Antiretroviral Therapy Outcomes

**DOI:** 10.3390/v6103855

**Published:** 2014-10-20

**Authors:** Allison Langs-Barlow, Elijah Paintsil

**Affiliations:** 1 Department of Pediatrics, Yale University School of Medicine, 333 Cedar St, New Haven, CT 06520, USA; E-Mail: allison.Langs-barlow@yale.edu; 2 Departments of Pediatrics and Pharmacology, Yale University School of Medicine, Child Health Research Center, 464 Congress Ave, New Haven, CT 06520, USA

**Keywords:** human immunodeficiency virus, subtypes, polymorphisms, mutations, drug resistance, antiretroviral therapy

## Abstract

Worldwide circulating HIV-1 genomes show extensive variation represented by different subtypes, polymorphisms and drug-resistant strains. Reports on the impact of sequence variation on antiretroviral therapy (ART) outcomes are mixed. In this review, we summarize relevant published data from both resource-rich and resource-limited countries in the last 10 years on the impact of HIV-1 sequence diversity on treatment outcomes. The prevalence of transmission of drug resistant mutations (DRMs) varies considerably, ranging from 0% to 27% worldwide. Factors such as geographic location, access and availability to ART, duration since inception of treatment programs, quality of care, risk-taking behaviors, mode of transmission, and viral subtype all dictate the prevalence in a particular geographical region. Although HIV-1 subtype may not be a good predictor of treatment outcome, review of emerging evidence supports the fact that HIV-1 genome sequence-resulting from natural polymorphisms or drug-associated mutations-matters when it comes to treatment outcomes. Therefore, continued surveillance of drug resistant variants in both treatment-naïve and treatment-experienced populations is needed to reduce the transmission of DRMs and to optimize the efficacy of the current ART armamentarium.

## 1. Introduction

Human immunodeficiency virus-1 (HIV-1) is a single-stranded RNA retrovirus with an inherent propensity towards sequence variation. At any given time, an HIV-infected individual harbors a heterogeneous population of HIV-1 referred to as quasispecies [1]. This genetic diversity is a consequence of the rapid replication rate of HIV-1, the tendency of HIV-1 reverse transcriptase (RT) to misincorporation of nucleotides and/or extend mispaired template-primer, and the evasion of the host immune system by the virus [2,3,4]. Moreover, the use of antiretroviral therapy (ART) adds selective pressure that favors the emergence of drug resistant variants, hence the possibility for transmission of HIV harboring drug resistant mutations, *i.e.*, transmitted drug resistance mutations (TDRMs) [5].

Four distinct genetic groups of HIV-1 exist worldwide: M (major), O (outlier), N (non-M, non-O), and P (new group) [2,6]. The most predominant group of HIV-1, M, is itself divided into nine subtypes (referred to as clades A-D, F-H, and J-K), 58 circulating recombinant forms (CRFs), and many unique recombinant forms (URFs). Furthermore, within each of the subtypes, CRFs, and URFs, there also exist genetic sequence variations that occur without the influence of antiretroviral pressure. These naturally occurring variations are referred to as polymorphisms.

Sequence variations that result from drug selection pressure are referred to as drug resistance mutations (DRMs). Therefore, DRMs are antiretroviral drug class dependent. Resistance to nucleoside analogs could result from either a single mutation or combinations of mutations in HIV-1 RT gene. A high level of resistance to zidovudine (AZT) or stavudine (d4T) results from accumulation of mutations in the RT (e.g., 41L, 67N, 70R, 210W, 215Y/F, and 219Q/E) [7,8]. These mutations are referred to as thymidine associated mutations (TAMs). In clinical isolates, two TAM pathways have been observed: 41L, 210W, 215Y/F and 67N, 70R, 219Q/E/N/R; of these, the 41-210-215 combination is the most prevalent [7,9]. The TAMs confer resistance to thymidine analogs by increasing RT’s phosphorolytic activity. RT with TAMs removes chain-terminating inhibitors from the 3’ end of the primer in the presence of physiological concentrations of pyrophosphate (PPi) or ATP [10,11,12]. The cytidine analogs (e.g., lamivudine–3TC and emtricitabine-FTC) select for the M184I/V mutation in RT, while the K65R is seen with tenofovir selection pressure. M184I/V and K65R mutations in RT confer resistance by altering discrimination between NRTIs and natural substrates (dNTPs) [10]. Certain combinations of RT mutations can result in resistance to multiple NRTIs. Notably are the Q151M complex and 69 insertion complex. The Q151M complex evolves by acquisition of Q151M mutation, followed by the mutations 62V, 75I, 77L, and 116Y [13]. The 69 insertion complex, consisting of a mutation at codon 69 (typically Ser), followed by an insertion of two or more amino acids (e.g., Ser-Ser, Ser-Arg, or Ser-Gly) as well as other nucleoside analog associated mutations (NAMs) [14]. With non-nucleoside reverse transcriptase inhibitors (NNRTIs), a single mutation (most commonly K103N or Y181C) confers high-level resistance to first-generation NNRTIs, such as nevirapine and efavirenz [15,16]. However, resistance to second-generation NNRTIs such as etravirine and rilpivirine results from multiple NNRTI-associated mutations [17,18]. The evolution of protease inhibitor resistance occurs in an ordered fashion: First, mutations developing, acting as “primary” resistance mutations confer inhibitor resistance; then “secondary” mutations develop that do not necessarily increase resistance but improve the replicative capacity of the virus [19,20]. Single or multiple mutations in the HIV-1 integrase gene can result in reduced efficacy of current integrase inhibitors. Several mutations have been identified in patients failing raltegravir containing regimens, including S230R, G163R, N155H, Q148K/R/H, Y143R/C/H, G140S/A, T97A, and L74M [21]. Resistance to elvitegravir is associated with the selection of one or more resistance mutations. Dolutegravir has a higher barrier to resistance than raltegravir and elvitegravir. Both *in vitro* and clinical data indicate that HIV-1 with primary mutations at codon 155 or 143, and the T66I and E92Q mutants remain susceptible to dolutegravir, whereas mutations at codon 148 in the presence of other secondary mutations (L74I/M, E138A/D/K/T, G140A/S, Y143H/R, E157Q, G163E/K/Q/R/S, or G193E/R) leads to decreased dolutegravir efficacy [22].

The primary goal of antiretroviral therapy is to suppress viral replication to undetectable levels as assessed by standard HIV RNA quantification assays. Viral suppression serves two main functions: immune restoration and the reduction of AIDS-related morbidity and mortality [23,24]. There are conflicting reports on the effect of DRMs on disease progression and death. While some studies report that DRMs are not predictive of morbidity [25,26]; others find a strong association between the two [27,28,29]. DRMs are typically not found in HIV treatment-naïve population; when they are, it is likely because of natural polymorphisms or transmitted drug resistant viruses. In addition, certain polymorphisms exist across HIV-1 subtypes that can confer varying levels of drug resistance or predispose an individual to developing DRMs [30]. Given that subtype B is the most prevalent in developed countries, it has been the major target of drug design. With the scale up of ART coverage in resource-limited settings, where non-subtype B HIV infections predominate, the questions that remain are: is there a difference in treatment outcomes between subtype B and non-B HIV subtypes; and what are the effects of polymorphisms and TDRMs on treatment outcomes? In this review, we focus on publications from the last 10 years that have looked specifically at HIV-1 sequence diversity, polymorphisms, and TDRMs as they pertain to clinical outcomes in HIV-infected populations receiving ART.

## 2. Effect of HIV Subtypes on Antiretroviral Therapy Outcomes

The majority of HIV-1 infections world-wide are due to non-subtype B virus. Fifty percent of all HIV-1 infections are actually attributed to subtype C, followed by A at 12%, and B at 10% [31]. Subtype B has traditionally been predominant in the Americas, Australia, and Western Europe. Subtype C is most common in sub-Saharan and East Africa, India, and Brazil. Subtype A is common in Eastern Europe and Asia. CRF01_AE is found in South East Asia, and CRF_02AG in West Africa [32]. Despite this, the anti-HIV activity of antiretroviral agents was discovered primarily based upon the life cycle of HIV subtype B. In addition, the effectiveness and evolution of resistance to current ART have mostly been described in developed countries with predominantly subtype B infection. Only in recent years has much effort and attention have turned to examining ART effectiveness and evolution of DRMs in non-B subtypes.

Although differences in drug susceptibility exist between subtypes, recent large international studies assessing effectiveness of various ART regimens have not demonstrated increased treatment failures in specific subtypes, although there have been instances of delayed CD4 cell count recovery [33,34,35,36] attributed to viral subtype. For instance, in a study conducted across Thailand, Hong Kong, Japan, Taiwan, and South Korea, 1036 individuals, of whom 778 harbored CRF01_AE virus and 258 individuals harbored sub-type B, were followed for 1547 person-years. Although CD4 counts were higher in the B *versus* non-B group, there were no differences in virologic response [36]. In contrast, a French study of 1413 subjects showed that individuals harboring CRF02_AG had a significantly better immune response to antiretroviral therapy compared with individuals infected with subtype B [35]. There was no difference in virologic response between subtypes. In a study from Israel, the authors noted that the perceived differences in treatment outcomes amongst various subtypes might actually be due to differences in quality of care provided across economic and geographic boundaries [33]. In their population, they showed that although there was a significant difference in rise of CD4 count by subtype, (175 in subtype B *versus* 98 in subtype C, *p* < 0.001), no such difference existed in viral loads or resistance rates. Interestingly, in a 2012 Italian study patients were matched across subtypes with the same resistance mutations; no difference in viral suppression was found by week 12 [37]. CD4 cell count recovery was not assessed in these patients. Lee *et al.* reported a faster immunologic decline in subtype CRF01_AE compared with subtype B, followed by a comparable slower and less robust CD4 count recovery upon initiation of antiretroviral therapy [38].

These findings corroborate ours and that of other investigators in sub-Saharan Africa who have demonstrated the effectiveness of ART in HIV-infected children and adults with predominantly non-B subtypes [39,40,41,42,43]. Taken together, one might conclude that markers such as resistance mutations, access to and quality of care, and pre-therapy CD4 counts might influence treatment outcomes more robustly than HIV-1 subtype.

The variation in intra-subtype drug susceptibility seems to stem from polymorphisms that have a higher prevalence within specific subtypes, such as non-B subtypes [17]. A well-documented example is that subtype C has a higher prevalence of the K65R mutation compared with subtype B [44]. However, not all individuals with subtype C will develop the K65R mutation; thus suggesting that a polymorphism within the subtype is responsible for the increased appearance of K65R under drug pressure [45]. Although at the individual level, being infected with one subtype *versus* another might impact the probability of developing a particular drug resistance, at the population level, no difference in treatment outcomes has been demonstrated among the subtypes. Thus, the best method for determining drug susceptibility still appears to be genome sequencing to look for specific drug resistance mutations or polymorphisms in individual patients; subtype alone is not predictive of drug treatment success or failure.

## 3. Effect of Polymorphisms on Antiretroviral Therapy Outcomes

There are conflicting reports at the population level as to the effect of polymorphisms on treatment outcomes. For instance, the Swiss Cohort Study did not show any difference in time to virological failure or time to virological suppression between HIV infected patients with 0 *versus* ≥ 1 minor polymorphisms in the protease gene [46]. In contrast, Mackie *et al.* showed that 57% of HIV infected patients in their London cohort had at least one polymorphism in the reverse transcriptase gene, and that those with ≥ 2 polymorphisms had significantly higher rates of virologic failure [47]. Thus, it stands to reason that variables such as geographic location, HIV-1 subtypes in circulation, and the number and location of polymorphisms all play a role in the outcomes associated with ART.

Polymorphisms are of interest when they directly impact either viral fitness or antiretroviral susceptibility. Interestingly, most of the major drug-resistance mutations (DRMs), as outlined by the Stanford HIV Resistance Database are not considered polymorphisms; they are only rarely, if ever, identified in drug-naïve populations of HIV-infected individuals [32]. The Stanford Drug Resistance Database is constantly updated with polymorphisms associated with HIV-1 drug resistance (for more details on polymorphisms and their significance refer to [48]).

### Mechanisms of Resistance by Polymorphisms

Naturally occurring HIV-1 variants, polymorphisms, confer resistance or improve viral replicative fitness through multiple mechanisms. For example, DRMs can reduce the replication capacity of the virus in addition to rendering it more resistant to a particular drug class [49]. However, when these mutations occur in the presence of polymorphisms or minor drug mutations, replicative fitness can often be restored. Furthermore, polymorphisms causing a change of a single nucleotide that results in a codon coding for the *same* amino acid may increase the probability of a virus developing DRMs by altering the way in which reverse transcriptase moves along the reading frame [45]. Polymorphisms can also reduce the genetic barrier to the development of resistance for various drug classes [48,49]. We will highlight some examples of the various ways in which specific polymorphisms contribute to antiretroviral resistance.

Some polymorphisms only confer resistance in the presence of other DRMs. For example, M50I often appears in infected cell cultures exposed to integrase inhibitors following the emergence of the R263K mutation. This combination confers resistance to dolutegravir, a second-generation integrase inhibitor, and has been observed in patients failing treatment with first generation-integrase inhibitors [50]. However, M50I also occurs naturally in approximately 10%–25% of integrase inhibitor naïve patients [51]. Alone, it does not appear to confer resistance; however, in combination with R293K, it confers moderate resistance to integrase inhibitors *in vitro* [50]. Another notable example is the combination of the V106I and V179D polymorphisms of reverse transcriptase that may confer resistance to efavirenz and nevirapine. Alone, V106I and V179D only confer a 1.5-fold increased NNRTI resistance compared to wild type virus; in combination they confer a 15-fold increased resistance [52]. One mechanism by which combinations of mutations and polymorphisms may complement one another is through the bolstering of replicative fitness which has been lost due to mutation. For example, Huang *et al.* showed that mutations at position 190 led to the production of virions with incompletely processed Gag-Pol protein, leading to downstream inefficiency of both protease and reverse transcriptase. It was observed that the presence of a L74V polymorphism increased replicative fitness in those viruses containing a G190V/E mutation (conferring resistance to nevirapine and/or efavirenz) by potentially improving the processing of Gag-Pol and increasing the stability of reverse transcriptase [53].

Polymorphisms at drug target sites may affect drug susceptibility. There is wide variability in the susceptibility of HIV-1 viral isolates to entry inhibitors; up to a 1000-fold difference in 50% inhibitory concentrations have been demonstrated [54]. Much of this variation is attributed to the highly variable viral envelope, the target for this class of drugs. Moreover, some of the mutations selected for during *in vitro* drug resistance studies using subtype B isolates are natural polymorphisms in non-B subtypes [55]. M426L, a mutation in HIV-1 gp120 that confers an 81-fold increased resistance to an entry inhibitor in development (BMS-626529), is present in 46% and 7% of patients with HIV-1 of subtype D and CRF01_AG, respectively. Most acquired mutations and natural polymorphisms alter the binding of the drug to its active site. Specifically, nearly all the mutations/polymorphisms studied (M36I, I15V, D30N, K45R, T74S, N88D, and L89V, L63P, E35D, H69K, K20T, and L90M) altered the binding site of the protease to an “open” flap configuration from its former “closed” flap configuration; hence decreasing the enzyme’s affinity for the drug [56].

Polymorphisms at the active site may influence the catalytic efficiency of the enzyme. In some circumstances, increased enzyme activity or expression is enough to overcome the drug effect. Ghosn *et al.* reported that polymorphisms found in the nucleotides encoding the Gag-Pol protein cleavage sites (the targets of protease) affected susceptibility to protease inhibitors (PIs) [57]. Interestingly, they found that non-B subtypes had higher rates of *gag* polymorphisms, especially in the p2/NC cleavage site. Furthermore, non-B subtypes were more likely to have more than two polymorphisms at this site; these changes were associated with virologic failure while on a protease inhibitor regimen.

Polymorphisms do not always confer antiretroviral resistance directly. Occasionally, the effect of a polymorphism on a viral enzyme simply increases the risk of developing broad antiretroviral resistance. For instance, HIV-1 subtype C has higher rates of the K65R major mutation which confers multidrug resistance to reverse transcriptase inhibitors. Coutsinos *et al.* demonstrated that the higher rate of the K65R mutation in subtype C is due to polymorphisms at codons 64 and 65 that change the more common AAG-AAA sequence (subtype B) to an AAA-AAG (subtype C) [45]. All three codons code for the same amino acid, lysine, but it appears that long runs of adenosine cause reverse transcriptase to pause during transcription. This pause leads to a higher rate of mutation at codon 65 in the subtype C viruses. Thus, AAA-AAG becomes AAA-AGG, transforming the lysine to an arginine at codon 65, and conferring resistance to the approved NRTIs [58].

Moreover, polymorphisms sometimes simply reduce the number of mutations it takes to for an individual virus to develop a major resistance mutation, *i.e.*, reducing the genetic barrier. For example, “AAG” (lysine) to “AGG” (arginine) requires one nucleotide change. However for “AAA” (lysine) to become “AGG” (arginine), requires two nucleotide substitutions. Thus, “AAA” has a higher genetic barrier to the development of the K65R mutation than “AAG” [58]. Martinez-Cajas *et al.* examined the mutational pathway to protease inhibitor resistance in non-B subtypes and found that M89L (ATG→ACG) is present in >95% of HIV-1 subtypes A, CRF01_AE, CRF02_AG, and G in addition to > 80% of subtype C worldwide [59]. When comparing HIV-1 sequence databases in various countries, these subtypes had higher rates of the M89I/V/T mutations that were further associated with failing protease inhibitor regimens.

## 4. Transmitted Drug Resistant Mutations

TDRMs are the current menace in HIV therapeutics. TDRMs could reverse the gains of ART, particularly during the unprecedented rollout in resource-limited countries. TDRMs can persist for several years with debatable effects of treatment outcomes [30,60,61,62]. The increasing and varying prevalence of TDRMs will limit antiretroviral regimen options for treatment-naïve patients. In addition, factors such as geographic location, access and availability of ART, duration since inception of treatment programs, quality of care, risk-taking behaviors, mode of transmission, and viral subtype all influence the prevalence in a particular locale [63,64]. In resource-rich countries, pre-HAART treatment options contributed to the rapid raise in the prevalence of TDRMs to as high as 27% [65,66,67]. As illustrated in Table 1, prevalence of TDRMs is proportional to the duration of availability of ART in a given locale. For instance, the introduction of ART in Southeast Asia is fairly recent; therefore, the prevalence of TDRMs is relatively low there. TDRMs in ARV-naive Africans are considered uncommon [68,69,70]. However, Price *et al.* recently reported significant variability in the overall prevalence of TDRMs across African study populations with an increasing prevalence over time (<5% to >15%) [71]. The reported prevalence of TDRMs in Africa is gradually mirroring that of TDRMs in resource-rich countries, with a range of 8% to 27% and increasing over time [65,72].

**Table 1:** Prevalence of drug resistance mutations in HIV treatment-naïve patients worldwide.

Continent	Country of Study	Prevalence of TDRM * (%)	Reference(s)
Africa	Mali	0%	[73]
	Cote d’Ivoire	6%	[69]
	Cameroon	<1%	[68]
	Uganda	3%–19%	[70,71]
	Tanzania	14.8%	[74]
	Rwanda	5%-15%	[71]
	Zambia	15.8%	[71]
	South Africa	<5%–20%	[71,75]
Asia	China	0%–12.5%	[76]
	Korea	13.6%–45.5%	[76]
	Japan	10.7%	[77]
Australia	Australia	13.4%–21.9%	[78,79]
Europe	Estonia	0%	[80]
	Italy	18.3%	[81]
North America	Caribbean †	0%	[82]
	United States	4.9%–24.9%	[83,84]
South America	Brazil	0%–15.4%	[85,86]

Adapted and modified from [48]; * TDRMs, transmitted drug resistant mutations; † Caribbean countries included in the study are Antigua, Dominica, Grenada, Guyana, Montserrat, St. Kitts, St. Lucia, St. Vincent, Suriname, Trinidad and Tobago; Prevalence of TDRMs tends to be higher in areas that had early access to antiretroviral medications. Countries highlighted are those that have reported the highest and lowest prevalence of drug resistance HIV-1 in treatment-naïve populations for each continent. For more detailed information on TDRM prevalence within other specific countries, please visit [48].

TDRMs are of public health concern particularly in resource-limited countries where most HIV-infected individuals are initiated on ART when CD4 counts fall below 350 cells/μL. Thus, at the time of initiation of treatment, TDRMs might have become minority variants. Minority variants constitute less than 15% of the viral population and, therefore, allele-specific sequencing assays are more sensitive for detecting the variants than bulk or population sequencing assays [87]. Furthermore, the specific resistance reference database used may influence the prevalence of DRMs observed. Ong *et al.* reported that using the WHO consensus guidelines, DRMs were not observed over a 5-year period in an HIV treatment-naïve cohort in Kuala Lumpur [88]. However, when they analyzed the sequences of this cohort against the Stanford guidelines, 35% of them had at least one mutation capable of reducing susceptibility to PIs, NRTIs, or NNRTIs.

Although Scherrer *et al.* found no association between the presence of minor PI DRMs at baseline and treatment outcome (e.g., time to virologic failure, time to viral suppression, or emergence of major PI mutations) in the Swiss HIV Cohort [46]. However, pre-existing DRM variants have been associated with treatment failure in multiple other studies [87,89,90]. In these studies, individuals infected with DRMs show a longer time to viral suppression and a shorter time to virologic failure after initiating ART compared with individuals infected with wild type viruses [91]. TDRMs have been associated with increased risk of poor virologic response after initiation of ART [89,92,93,94]. In a pooled analysis of 985 participants from 10 studies, the presence of minority DRMs was associated with a 2–3 fold higher risk of virologic failure after initiating ART in treatment-naïve patients [95]. In a study from sub-Saharan Africa which enrolled over 2500 HIV-infected individuals between 2007 and 2009, 5% of the patients had at least one pre-treatment DRM directed to at least one component of their antiretroviral regimen. Of these, only 75% achieved virologic suppression at 12 months of treatment compared to 91% virologic suppression in those without pretreatment DRMs [96]. This finding is consistent with an earlier observation in a European cohort in which the presence of TDRMs also reduced virologic response to ART [97]. Cambaino *et al.* recently used a mathematical model to estimate the potential long-term impact of TDRMs on mortality [98]. This model took account of the loss of mutations during the transmission process or over time in the host. Outcomes were compared over 45 years from baseline, taking into consideration a scenario in which there was no change in the ability of the resistant virus to be transmitted, and a second scenario in which transmission of resistant virus was not possible from baseline onwards. It was shown that epidemics with higher levels of TDRMs at baseline tend to continue to have higher levels of TDRMs throughout. Moreover, it was observed that the long-term impact of moderate to high levels of TDRMs on mortality could be substantial [98].

## 5. Conclusions

Appreciation of HIV-1 sequence diversity is a critical element for drug discovery and for optimization of ART, especially in drug-naïve patients. Although HIV-1 subtype may not be a predictor of treatment outcome, review of emerging evidence supports the fact that natural polymorphisms or drug-associated mutations matter when it comes to treatment outcomes. HIV-1 continues to evolve rapidly, thus introducing ever more complex sequence diversity. Therefore, continued surveillance of DRMs in both treatment-naïve and treatment-experienced populations is needed to reduce the transmission of DRMs and to optimize the efficacy of the current antiretroviral armamentarium. An ideal treatment algorithm would include the assessment of a patient’s HIV-1 genotype prior to ART initiation. However, this is not technically and economically feasible at the present time, particularly in resource-limited countries. ART programs in resource-limited countries have adopted the WHO HIV drug resistance guidelines: a population-based survey of transmitted HIV drug resistance in recently infected individuals [99]. The WHO recommends three key assessment elements of a country’s prevention strategies regarding HIV drug resistance: (1) routine monitoring of factors known to be associated with the emergence of HIV drug resistance at site and program levels, *i.e.*, HIV drug resistance “Early Warning Indicators” (EWI) [100]; (2) surveys to assess transmitted HIVDR in recently infected populations [101]; and (3) surveys to monitor the emergence of HIVDR [83] and related programmatic factors in populations receiving ART [102]. The prevalence of transmitted drug resistant HIV in a specific geographic area is classified as: low prevalence: <5%; moderate prevalence: 5%–15%; or high prevalence: >15%. These drug resistance surveys are designed to generate data that will inform evidence-based decisions regarding the future selection of national and global ART regimens. Even in resource-rich countries, where genotyping prior to initiation of treatment is included in the treatment guidelines [103], there are challenges regarding the choices among genomic assays (population sequencing, single-genome sequencing, or ultradeep pyrosequencing) as well as the difficulties related to translation of the sequence data into treatment regimens [104]. As the HIV genome continues to evolve, and our knowledge of its ability to evade current therapies increases, our treatment algorithms must also adapt that we might continue to offer the best treatment options for our patients, regardless of HIV subtype.
